# Correlation Assisted Strong Uncorrelating Transform Complex Common Spatial Patterns for Spatially Distant Channel Data

**DOI:** 10.1155/2018/4281230

**Published:** 2018-05-15

**Authors:** Youngjoo Kim, Jiwoo You, Heejun Lee, Seung Min Lee, Cheolsoo Park

**Affiliations:** ^1^Department of Computer Engineering, Kwangwoon University, Seoul 01897, Republic of Korea; ^2^School of Electrical Engineering, College of Creative Engineering, Kookmin University, Seoul 02707, Republic of Korea

## Abstract

The Strong Uncorrelating Transform Complex Common Spatial Patterns (SUTCCSP) algorithm, designed for multichannel data analysis, has a limitation on keeping the correlation information among channels during the simultaneous diagonalization process of the covariance and pseudocovariance matrices. This paper focuses on the importance of preserving the correlation information among multichannel data and proposes the correlation assisted SUTCCSP (CASUT) algorithm to address this issue. The performance of the proposed algorithm was demonstrated by classifying the motor imagery electroencephalogram (EEG) dataset. The features were first extracted using CSP algorithms including the proposed method, and then the random forest classifier was utilized for the classification. Experiments using CASUT yielded an average classification accuracy of 78.10 (%), which significantly outperformed those of original CSP, Complex Common Spatial Patterns (CCSP), and SUTCCSP with *p*-values less than 0.01, tested by the Wilcoxon signed rank test.

## 1. Introduction

Noninvasive measurements of physiological signals including electroencephalogram (EEG), electrocardiogram (ECG), and electromyogram (EMG) have become widely used throughout the biomedical industry [1–5]. Out of the various feature engineering methods, researchers have shown that the common spatial patterns (CSP) algorithm is a strong feature extraction algorithm for multichannel EEG data, yielding high performance for classification problems [6, 7]. CSP is a mathematical methodology to decompose spatial subcomponents of multivariate signals, whose variance difference between two classes is maximized [8]. CSP designs spatial filters for the multichannel EEG signals based on the spatial distribution of neural activities in the cortex areas [6, 7] and adopts a supervised learning approach, while the other spatial filter algorithms such as principal component analysis (PCA) and independent component analysis (ICA) are designed in an unsupervised manner [9, 10].

Furthermore, a complex version of CSP, termed CCSP, uses the covariance matrix that maintains the power sum information of the real and imaginary parts of the complex-valued data [11]. Another complex-valued CSP algorithm, termed analytic signal-based CSP (ACSP), was proposed by Falzon et al. to discriminate different mental tasks [12, 13]. However, given that the Hilbert transformed analytic signals could only produce circular signals (rotation invariant probability distribution) and that physiological signals are improper (mismatch of power between different channel data), the augmented complex CSP was introduced to fully exploit the second-order statistics of noncircular complex vectors [11, 14].

Strong Uncorrelating Transform CCSP (SUTCCSP), which is an advanced version of the augmented complex CSP, was applied to the two-class classification problem of motor imagery EEG and produced a minimum of 4% improvement over the conventional CSP, ACSP, and augmented CSP [11]. This is due to the power difference information preserved in the pseudocovariance matrix, accompanied with the sum of power maintained in the covariance matrix. However, during the simultaneous diagonalization process of the covariance and pseudocovariance matrices, the correlation term vanishes owing to the process of applying the strong uncorrelating transform [11, 15, 16]. Such effort to preserve correlation has not been made so far for the CSP algorithm, and the correlation assisted version of SUTCCSP is newly proposed in this paper.

The basic terminologies and procedure of SUTCCSP and the proposed method are explained in Section 2, followed by extensive simulation results on the benchmark motor imagery dataset of 105 subjects in Section 3. Finally, the concluding remarks are given in Section 4 with additional discussions in terms of the performance difference of distinct channel pairs that have less correlation compared with results of Section 3.

## 2. Proposed Method

Here we explain SUT based on the terminologies used in [9, 14] and show how the correlation information is utilized with CSP algorithms [11, 16].

Let *x* be a complex-valued random vector such as

(1) $$\begin{array}{c} x=x_{r}+jx_{i}, \end{array}$$

where *j* is √(−1), *x*_*r*_ is the real part, and *x*_*i*_ is the imaginary part of a complex random vector. **X**_*k*_ is a zero-mean complex-valued matrix consisting of values with the form of (1), where *k* denotes the two different classes, *k* ∈ {1, 2}. **X**_*k*_ has the dimension of the number of channels by the number of samples. Then the covariance (**C**) and pseudocovariance (**P**) matrices are defined as follows:

(2) Ck=EXkXkH,Pk=EXkXkT,

where *E*(·) is the statistical expected value operator and (·)^H^ is the conjugate transpose. Then, we can define the composite covariance (**C**_*c*_) and pseudocovariance (**P**_*c*_) matrices as follows:

(3) Cc=∑kCk=EX1X1H+EX2X2HPc=∑kPk=EX1X1T+EX2X2T.

Here **C**_*c*_ can then be decomposed into Θ_*c*_ and Λ_*c*_ as follows:

(4) $$\begin{array}{c} C_{c}=\Theta_{c}\Lambda_{c}\Theta_{c}^{H}=\Theta_{c}\Lambda_{c}^{1/2}\Lambda_{c}^{1/2}\Theta_{c}^{H}, \end{array}$$

where Θ_*c*_ has eigenvectors in each column for the corresponding diagonal eigenvalues of Λ_*c*_. Note that Θ_*c*_ and Λ_*c*_ consist of real elements and the nondiagonal elements of Λ_*c*_ are zero. This allows **C**_*c*_ to be whitened by the whitening matrix Φ = Λ_*c*_^−1/2^Θ_*c*_^H^ in the original CCSP algorithm, resulting in Φ**C**_*c*_Φ^H^ = **I**, where **I** denotes the identity matrix [11].

Using the whitening matrix Φ = Λ_*c*_^−1/2^Θ_*c*_^H^ from the original CCSP algorithm [11], the pseudocovariance matrix can also be decomposed using Takagi's factorization as shown in the following equation [17]:

(5) $$\begin{array}{c} \Phi P_{c}\Phi^{T}=\Delta \Lambda \Delta^{T}, \end{array}$$

where Δ and Λ are yielded by symmetric matrices. This leads to a derivation of the strong uncorrelating transform matrix **S** as follows:

(6) $$\begin{array}{c} S=\Delta^{H}\Phi . \end{array}$$

Using the matrix **S**, it is now possible to diagonalize the covariance and pseudocovariance matrices simultaneously. Firstly, the composite covariance matrix can be diagonalized as follows:

(7) SCcSH=SC1SH+SC2SH=M1+M2=I∵Mk=SCkSH,  k∈1,2Y−1MkY=Λk,∑kΛk=I,

where **Y** and Λ are the estimations of eigenvectors and eigenvalues of **M**, respectively. Next, the composite pseudocovariance can also be diagonalized as follows:

(8) SPcSH=SP1SH+SP2SH=ΛS^=Λ−1/2ΔHΦM^k=S^PkS^TS^PcS^T=∑kM^k=IY^−1M^kY^=Λ^k,∑kΛ^k=I,

where $\hat{S}$ is the strong uncorrelating transform matrix for the pseudocovariance and $\hat{Y}$ and $\hat{\Lambda }$ are the estimations of the eigenvectors and eigenvalues of $\hat{M}$, respectively. Therefore, the two spatial filters **W** and $\hat{W}$ can be designed as follows:

(9) W=Y−1SW^=Y^−1S^.

Finally, the spatially filtered vector, **Z**, is calculated as follows:

(10) $$\begin{array}{c} Z=WX. \end{array}$$

Let *N* be the number of data channels, and *z*_*p*_ the *p*th row vector in **Z**;

(11) $$\begin{array}{c} Z^{\prime }=\left[\begin{array}{c} \begin{array}{c} z_{1}\\ \begin{array}{c} \begin{array}{c} \vdots \\ z_{m} \end{array}\\ \begin{array}{c} z_{N-m+1}\\ \vdots \end{array} \end{array} \end{array}\\ z_{N} \end{array}\right]=\left[\begin{array}{c} z_{1}^{\prime }\\ \vdots \\ z_{2m}^{\prime } \end{array}\right], \end{array}$$

where *z*_*p*_′ corresponds to each row of the new matrix **Z**′. Now the final subfeatures, *f*_*p*_ and *f*_*p*_′, by SUTCCSP are calculated as follows:

(12) fp=log⁡var Rzp′∑pvar Rzp′fp′=log⁡var Izp′∑pvar Izp′,

where *p* varies between 1 and 2*m* and var(·) is the variance of (·). Here, selecting one pair of filter is equivalent to choosing the first and last rows in each real and imaginary part of the covariance and pseudocovariance matrices, separately. The number of filter pairs was chosen to maximize the performance for each subject. Such consideration of selecting the appropriate number of filter pairs could be important in real time applications. Next, Pearson's correlation coefficient for *x*_*r*_ and *x*_*i*_ is calculated as follows [17]:

(13) $$\begin{array}{c} \rho_{x_{r},x_{i}}=\frac{E\left[\left(x_{r}-\mu_{x_{r}}\right)\left(x_{i}-\mu_{x_{i}}\right)\right]}{std\left(x_{r}\right)std\left(x_{i}\right)}, \end{array}$$

where std(·) is the standard deviation of (·) and *μ*_*x*_ is the mean of *x*. The maximum number of correlation coefficients between the real and imaginary parts of (1) is equal to the number of channel pairs due to the multichannel attribute of the data. The high dimension of the number of channel pairs should be reduced owing to the curse of dimensionality. PCA is applied to reduce the high dimension in this paper, due to its simple implementation and fast speed [18, 19].

Let Γ be the matrix containing *ρ*_*x*_*r*_,*x*_*i*__ for *N*(*N* − 1)/2 channel pairs, where *N* is the number of channels. By applying PCA to the correlation coefficient matrices, the principal component coefficients, known as loadings, are estimated [20]. Here we will define Ψ as an *N*-by-*L* matrix of loadings, where* L* is the reduced number of dimensions. An additional subfeature *f*_*q*_′′ containing the correlation information of two data channels is calculated as follows:

(14) $$\begin{array}{c} f_{q}^{\prime \prime }=\Gamma \Psi \;\left(q=1,\ldots ,L\right). \end{array}$$

The final feature matrices for two different classes are *f*_*p*_, *f*_*p*_′, and *f*_*q*_′′ for each class. In this paper, the covariance matrix information from the original CSP is added to the feature matrices of CCSP, SUTCCSP, and CASUT, which could provide a fair test to compare CSP with these three algorithms. Accordingly, the feature matrices of CASUT were designed to contain the information of variance, power sum, and difference, as well as the correlation information lost due to the strong uncorrelating transform.

## 3. Experiments

### 3.1. Data Acquisition

As Park et al. used the Physiobank Motor Mental Imagery (MMI) database to test the performance of SUTCCSP, this study uses the same dataset in order to compare the proposed CASUT with the former CSP algorithms including SUTCCSP [11, 21–23]. Out of the 109 subjects who conducted the left- and right-hand motor imagery tasks, three subjects (S088, S092, and S100) had damaged recordings, and one subject (S104) had an insufficient amount of data [15, 24]. For these reasons, 105 subjects were used to examine the classification accuracy of CASUT. All subject data consist of 45 trials of performing the left- and right-hand tasks, which were recorded using 64 channel electrodes with the 10-10 EEG system and sampled by 160 Hz [25].

In order to verify the performance of CASUT in preserving the correlation information, the channel pairs that yield high correlation coefficients were selected (values over 0.9 and less than or equal to 1). All trials for the left-hand motor imagery task of 105 subjects were combined into one single trial set, and the correlation coefficients of the all possible distinct 2016 pairs among the 64 channels were calculated. Then the average of the correlation coefficient values over all trials of the left-hand task was calculated, in order to determine which channel pair has a high correlation coefficient. The same calculation was conducted on the trials of the right-hand motor imagery task. The channel pairs, whose correlations were in the range of the following equation, were denoted as

(15) $$\begin{array}{c} r_{t}=\left\{\;\left(\;x,y\;\right)\;|\;t\cdot 10^{-1}<\rho_{x_{r},x_{i}}\leq \left(\;t+1\;\right)\cdot 10^{-1}\;\right\}, \end{array}$$

where (*x*, *y*) is a pair of two distinct channels *x* and *y*, *ρ*_*x*_*r*_,*x*_*i*__ are the correlation coefficients between *x* and *y*, and *t* is a number in the range of 0 ≤ *t* ≤ 9.

The EEG recordings were preprocessed using the fifth-order Butterworth IIR bandpass filter extracting the frequency components into 8–25 Hz [6, 26, 27]. Such preprocessing techniques were identical to the preprocessing techniques used by Park et al. [11].

### 3.2. Classification Results

#### 3.2.1. Analysis of 105 Subjects

The average classification accuracies over all 105 subjects were calculated in order to compare the proposed algorithm with CSP, CCSP, and SUTCCSP.  Table 1 shows the average classification rates with the standard deviations for each algorithm. Note that the classification rate of CASUT outperforms those of CSP, CCSP, and SUTCCSP.

The normality was tested to determine whether to use the parametric or nonparametric version of a statistical test such as ANOVA. Accordingly, the resulting *p*-values of the Kolmogorov-Smirnov goodness-of-fit hypothesis test (KS test) in Table 2 show that the classification accuracies of CSP algorithms could not always satisfy the normality assumption [28]. Therefore, the nonparametric Friedman test was used instead of the parametric ANOVA, to compare three or more matched groups regardless of their normality [29, 30].

The *p*-value for the Friedman test, which was less than 10^−15^, indicates that it is safe to perform the post hoc test. Instead of the parametric paired Student's *t*-test, the Wilcoxon signed rank test, which can be used regardless of the normality, was conducted as the post hoc test [28]. Although the average classification accuracy difference between CASUT and SUTCCSP looked small, the Wilcoxon signed rank test performed on the accuracies of the two algorithms yielded significant *p*-values (<0.05), as shown in Table 3. The *p*-values, *p*_1_, *p*_2_, and *p*_3_, indicate the results of the Wilcoxon signed rank test conducted on the classification accuracies of CASUT compared with those of original CSP, CCSP, and SUTCCSP, respectively.

#### 3.2.2. Analysis of Significant Subjects.

**Figure 1:**
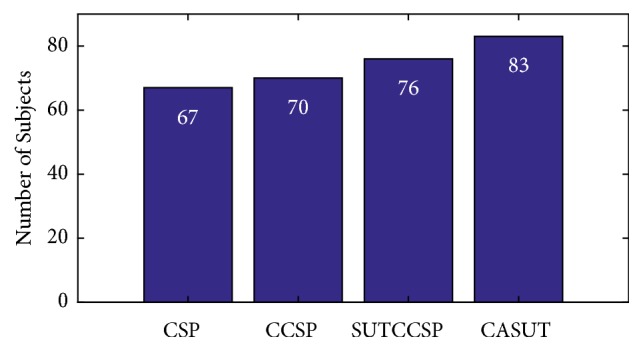
Number of significant subjects of CSP, CCSP, SUTCCSP, and CASUT. Note that the number of subjects for CASUT is the highest out of the four CSP algorithms.

For a thorough validation of the classification performances of the CSP algorithms, an additional analysis that was conducted by Park et al. was adopted by selecting the significant subjects prior to any further analysis [11]. This is crucial due to the possibility of little brain network information in the recorded EEG and activities of poorly performed subjects, based on the study of Ahn and Jun [31]. For these reasons, the subjects were categorized as significant, when the performance of each subject exceeded the minimum classification accuracy of 64%, defined using the confidence limit of 95% [32].  Figure 1 shows the number of significant subjects for each CSP algorithm. It can be observed that the number of significant subjects using CASUT was the highest out of all four CSP algorithms. The results throughout this chapter were based on the histograms of Figure 1, from which the data of the significant subjects were chosen for further analysis.

Table 4 lists the average classification accuracies over the significant subjects and their standard deviations for CSP algorithms. It can be also noted that the average classification rate of CASUT outperformed those of CSP, CCSP, and SUTCCSP. The KS test was also performed for the significant subjects. However, the results from Table 5 indicated that the classification accuracies of the CSP algorithm do not follow the normal distribution. Accordingly, the Friedman test, which can be used regardless of the normality, was conducted. The *p*-value from the Friedman test yielded a value less than 10^−12^, and thus the post hoc test was conducted and shown in Table 6. Note that the low *p*-values (<0.01) by the Wilcoxon signed rank test demonstrate the enhanced performance of CASUT.

**Figure 2:**
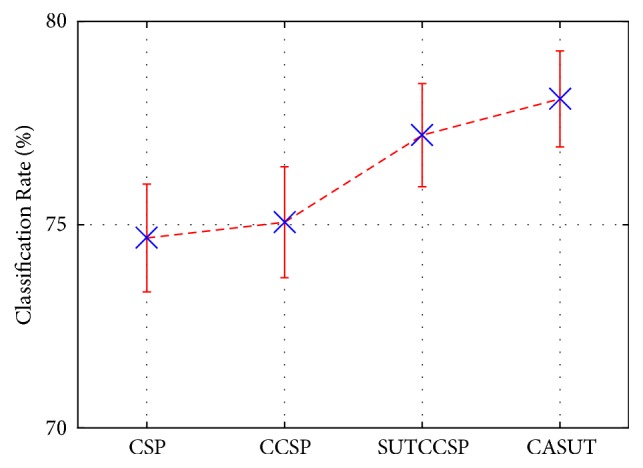
Error bar of the classification accuracies of CSP, CCSP, SUTCCSP, and CASUT. Note that CASUT produces higher classification rates compared with those of the other CSP algorithms, which is confirmed by the Wilcoxon signed rank test results of Table 6.

**Figure 3:**
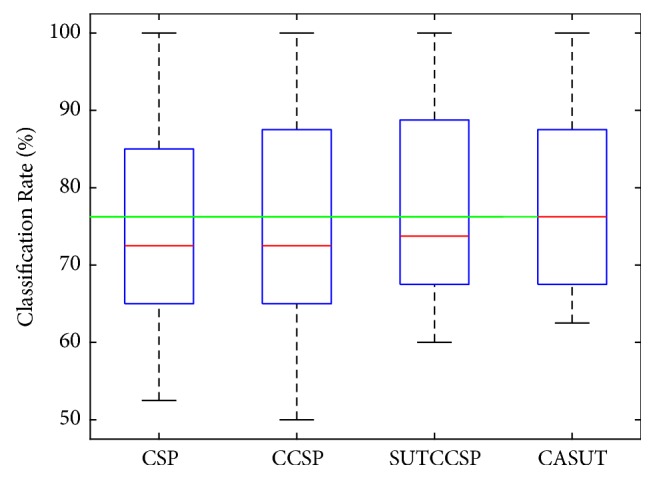
Whisker diagram of the classification accuracies of CSP, CCSP, SUTCCSP, and CASUT. The median of CASUT is highest compared with CSP, CCSP, and SUTCCSP.

Additional plots of the error bar and whisker diagram of the classification accuracies of CSP, CCSP, and CASUT were illustrated in Figures 2 and 3, respectively. The blue crosses in Figure 2 were identical to the average classification rates shown in Table 4. The red lines in Figure 3 indicate the median classification rates, and it can be observed that the median of CASUT outperforms those of the other three CSP algorithms. The superiority of CASUT over the other CSP algorithms was also confirmed by the Wilcoxon signed rank test results in Table 6.

**Figure 4:**
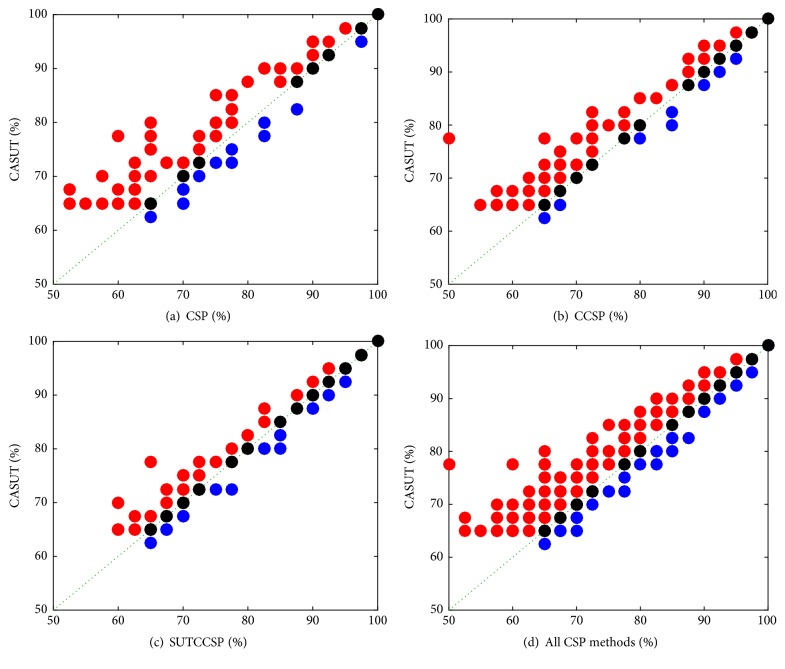
Scatterplot of classification rates of CASUT with (a) CSP, (b) CCSP, (c) SUTCCSP, and (d) the overlapping results of (a), (b), and (c). Note that most of the dots are located above the dotted green line, which indicates higher performance of CASUT.

In Figure 4, the scatterplots comparing classification rates of CASUT with CSP, CCSP, and SUTCCSP were displayed. The red dots above the dotted green lines indicate that classification rates were higher by CASUT than the other CSP algorithm. The black dots mean that CASUT and the compared CSP algorithm have the same classification rates, and blue means that the performances of CASUT are lower than those of the compared CSP algorithm. This demonstrates the fact that the majority of classification accuracies by CASUT were higher than those of the other CSP algorithms. Additionally, when two or more subjects yielded the same classification accuracies by two of the different algorithms, the dots for the subjects in these figures were duplicated. Therefore, the number of selected subjects in Figure 1 and the number of dots in Figure 4 may differ.

**Figure 5:**
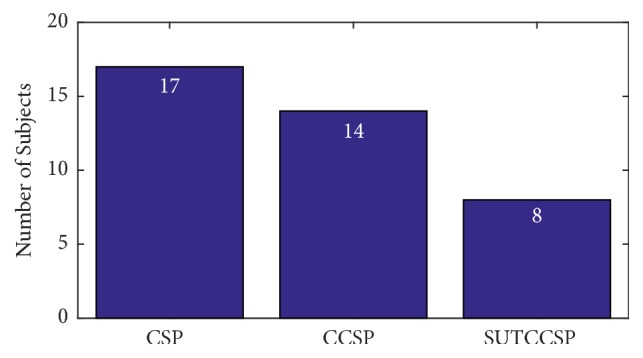
Number of subjects that were classified as significant with CASUT, but not with CSP, CCSP, and SUT, respectively.

Lastly, the number of subjects, classified significantly using CASUT and classified insignificantly using the other CSP algorithms, was counted and shown in Figure 5. The bar chart indicates the number of subjects that were classified as significant by CASUT, but not by CSP, CCSP, and SUT, respectively.

On the other hand, there was only one subject whose data was classified as insignificant by CASUT, while the other CSP algorithms classified it as significant. These results also demonstrate the superiority of CASUT over the other conventional CSP algorithms.

#### 3.2.3. Analysis of Correlation Assisted CSP

The various versions of CSP algorithms were additionally investigated for further interpretation of the effects of correlation information on the features of motor imagery tasks. To this end, correlation assisted CSP (CACSP) is defined as a CSP algorithm containing the correlation information, whereas correlation assisted CCSP (CACCSP) is defined as CCSP including the correlation information. The benchmark tests including CSP, CACSP, CCSP, CACCSP, SUTCCSP, and CASUT could provide an exact interpretation of the effects of correlation information on the features of the motor imagery tasks.

Table 7 lists the average classification rates calculated using CSP, CACSP, CCSP, CACCSP, SUTCCSP, and CASUT in the same conditions set in Table 4. Friedman test was conducted and a *p*-value less than 10^−15^ was confirmed. In Table 8, the Wilcoxon signed rank test was performed on CASUT with the other CSP algorithms, including CACSP and CACCSP. Results in bold show the results of the additional implementations of CSP and CCSP, that is, CACSP and CACCSP, respectively. Note that all *p*-values are significant, indicating the enhanced performance of CASUT over the others. Since CCSP contains the power sum information, additional to the CSP features, and SUTCCSP preserves the power difference information, supplementary to CCSP, the gradually increasing classification rates could be expected as shown in Table 4.

Similarly, the performances of CSP and CCSP increase as the correlation information is added to their original features. Additionally, the highest classification accuracy in these benchmark tests was yielded using CASUT, indicating that CASUT outperforms all former CSP algorithms introduced so far.

## 4. Discussion and Conclusion

**Figure 6:**
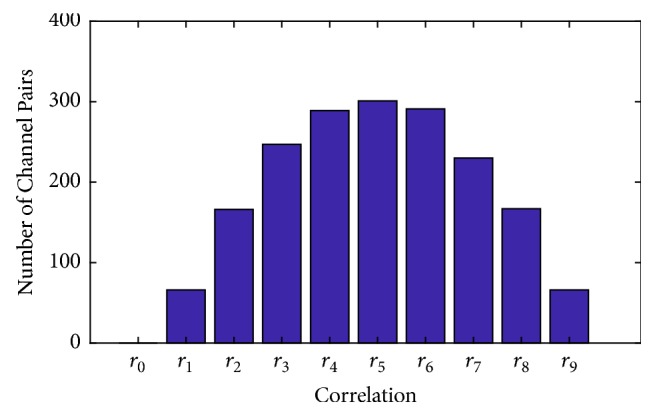
Number of channel pairs for each correlation range (*r*_0_ to *r*_9_).

**Figure 7:**
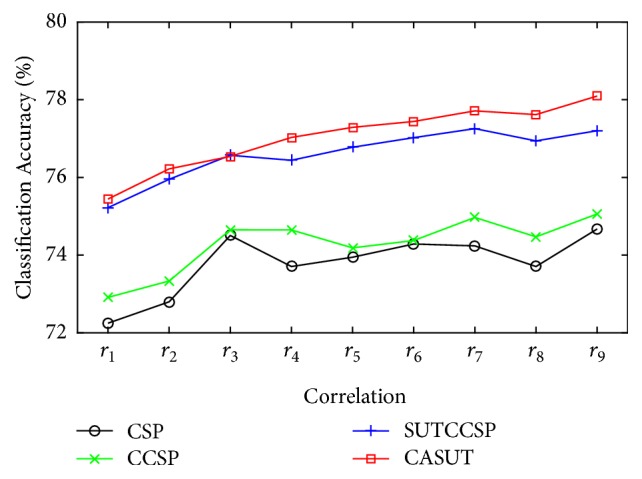
Classification accuracies for different correlation ranges (*r*_1_ to *r*_9_) of CSP, CCSP, SUTCCSP, and CASUT.

**Figure 8:**
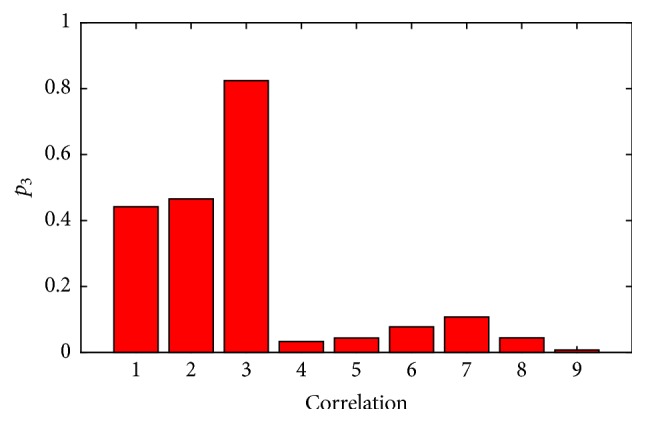
Resulting *p*-values of Wilcoxon signed rank tests conducted on CASUT with SUTCCSP for different correlation ranges (*r*_1_ to *r*_9_).

The correlation range chosen to evaluate the performance of CASUT was *r*_9_, based on (15). As shown in Figure 6, the number of channel pairs for each correlation range (*r*_0_ to *r*_9_) differs from zero to 301 channel pairs. In order to examine the effects of the correlation information on the CSP algorithms, the average classification accuracies over 105 subjects across different correlation ranges were calculated based on the same analysis in Section 3. Results demonstrate that the performance of CASUT gradually decreases as the correlation information is degraded as shown in Figure 7. Additionally, Figure 8 illustrates the resulting *p*-values estimated using the Wilcoxon signed rank test on CASUT compared with SUTCCSP, indicating less significance with small correlation coefficients. This proves that CASUT is the most effective feature extraction approach, when sufficient correlation information exists among the multichannel data.

The limitations of SUTCCSP have been addressed in this study due to the loss of the correlation information during the simultaneous diagonalization process of the covariance and pseudocovariance matrices. To that end, the correlation assisted version of SUTCCSP, denoted by CASUT, has been proposed for the first time by preserving the correlation information among multichannel data. The proposed algorithm was tested on the two-class motor imagery classification problem, and its classification accuracies obtained using the channel pairs with high correlation were significantly improved by CASUT compared with those of CSP, CCSP, and SUTCCSP, with *p*-values less than 0.01. Additional experiments on the various ranges of correlation prove that the correlation information is crucial to the classification of the two-class motor imagery tasks and that CASUT yields the highest classification accuracies compared with the other CSP algorithms.

## Figures and Tables

**Table 1:** Average classification accuracies of CSP, CCSP, SUTCCSP, and CASUT across 105 subjects.

CSP method	CSP	CCSP	SUTCCSP	CASUT
Classification accuracy (%)	70.62 ± 1.35	70.60 ± 1.41	73.05 ± 1.32	73.69 ± 1.30

**Table 2:** The resulting *p*-values of the KS test for each CSP algorithm for 105 subjects.

CSP method	CSP	CCSP	SUTCCSP	CASUT
*p*-values	0.1784	0.1568	0.0777	0.2533

**Table 3:** Results of the Wilcoxon signed rank test conducted on performance accuracies of CASUT compared with those of CSP, CCSP, and SUTCCSP using 105 subjects.

	*p*_1_	*p*_2_	*p*_3_
*p*-value	<10^−7^	<10^−10^	<0.05

**Table 4:** Average classification accuracies across the significant subjects of CSP, CCSP, SUTCCSP, and CASUT.

CSP method	CSP	CCSP	SUTCCSP	CASUT
Classification accuracy (%)	74.68 ± 1.33	75.06 ± 1.36	77.20 ± 1.27	78.10 ± 1.18

**Table 5:** The resulting *p*-values of the KS test for each CSP algorithm for significant subjects.

CSP method	CSP	CCSP	SUTCCSP	CASUT
*p*-values	0.2087	0.0359	0.0282	0.0418

**Table 6:** Results of the Wilcoxon signed rank test conducted on the classification accuracies of CASUT compared with those of CSP, CCSP, and SUTCCSP for significant subjects.

	*p*_1_	*p*_2_	*p*_3_
*p*-value	<10^−7^	<10^−8^	<0.01

**Table 7:** Average classification accuracies across 105 subjects of CSP, CASUT, CCSP, CACCSP, SUTCCSP, and CASUT.

CSP method	Classification accuracy (%)
CSP	74.06 ± 1.30
**CACSP**	**75.11 ± 1.33**
CCSP	74.18 ± 1.38
**CACCSP**	**74.83 ± 1.30**
SUTCCSP	76.45 ± 1.27
CASUT	77.36 ± 1.19

**Table 8:** Results of the Wilcoxon signed rank test conducted on performance accuracies of CASUT compared with those of CSP, CACSP, CCSP, CACCSP, and SUTCCSP.

	*p*-value
*p*_1_	<10^−7^
**P**_**CACSP**_	<**10**^-**5**^
*p*_2_	<10^−8^
**P**_**CACCSP**_	<**10**^-**7**^
*p*_*3*_	<0.01
